# Microhomology-mediated end-joining Knock-In approaches to delete the allergenic domain of trout *parvalbumin beta-1*. Preliminary results in F0 animals and feedback

**DOI:** 10.12688/openreseurope.18223.1

**Published:** 2024-10-25

**Authors:** Veronique Lebret, Cecile Duret, Amaury Herpin, Pierre-yves Rescan

**Affiliations:** 1 LPGP Fish Physiology and Genomics, Institut National de Recherche pour l'Agriculture l'Alimentation et l'Environnement Centre Bretagne-Normandie, Rennes, Brittany, 3542, France

**Keywords:** Gene editing, microhomology-mediated end joining Knock-In, parvalbumin, allergenicity, trout, Genetically modified organisms (GMOs).

## Abstract

**Background:**

Gene editing techniques offer new opportunities to improve important traits in aquaculture. The allergenicity of fish flesh is a major problem in aquaculture. Parvalbumin (Parv) is the most prevalent fish allergen. For instance, in salmonids, a single parvalbumin beta-1 protein (parvb1) has been identified as an allergen in specific patients. Therefore, generating trout carrying two parvb1 alleles deleted from the allergenic peptide-encoding region could prevent allergies in these sensitive individuals.

**Methods:**

Here, we describe the application of the Crispr/cas9 system in an attempt to delete parvb1 exon 2 encoding the allergenic peptide and, alternatively, to replace exon 2 of parvb1 with exon2 of parvalbumin beta-2 protein (parvb2,) which does not encode the allergenic peptide. Exon skipping and swapping were pursued through microhomology-mediated end-joining (MMEJ) knock-In using specifically designed double-stranded donor DNA.

**Results:**

Genotyping of approximately 200 F0 fingerlings originating from eggs injected with donor DNA designed for exon 2 skipping led to the identification of only one animal carrying an allele lacking exon 2. Genotyping of approximately 150 fingerlings originating from eggs injected with donor DNA for exon 2 swapping did not result in any trout carrying the expected modified allele.

**Conclusions:**

These preliminary results indicate the potential difficulties associated with the MMEJ KI experiments performed in farmed fish. Finally, new genomic techniques in aquaculture are further discussed in the context of lively debates taking place in the European parliament regarding a possible revision of the current law that determines the legal status of farm animals modified by genome editing.

Gene editing, microhomology-mediated end-joining knock-in, parvalbumin, allergenicity, trout, and genetically modified organisms (GMOs).

## Plain language summary

Unlike transgenesis, new gene-editing technologies allow site-specific genetic changes. These new technologies can be used either to invalidate a targeted gene or to precisely modify the nucleotide sequence of a gene of interest, allowing, for example, production of a reshaped protein. As such, gene editing may become a popular technique for developing new phenotypic traits of interest in farmed animals, especially in aquaculture species. Parvalbumin is a major allergen in fish. Conceptually, a simple way to suppress fish allergies would be to inactivate the gene encoding the allergen. However, due to its role in calcium binding, the complete knockout of parvalbumin could have major detrimental impacts on animal physiology, particularly with respect to muscle calcium signalling and contraction. Previous research has shown that only a small domain of the trout parvalbumin beta-1 protein is allergenic. Thus, a way to suppress fish allergies without affecting muscle physiology could be to remove, replace, or modify the allergenic domain of the protein through targeted modifications of the parvalbumin gene. In this study, we describe two CRISPR/Cas9-based editing strategies to generate trout with parvalbumin beta-1 alleles lacking an allergen-encoding subdomain. Although such a procedure could be considered as technically trivial, the success rate was at the end found to be very low. This suggests, at least in fish, that complex gene editing strategies definitely need further improvements before being routinely employed as popular procedures for engineering desirable phenotypic traits by means of allele modifications.

## Introduction

Aquaculture has become a type of animal food production with the fastest growth in recent decades ^1^. Fish are among the most important groups of allergenic foods worldwide ^2, 3^. Allergic reactions result in sensitized consumers from an excessive IgE-mediated response of the immune system against specific proteins called allergens. The major allergen responsible for fish allergies in humans is parvalbumin ^4^. Parvalbumins are heat-stable intracellular EF-hand proteins involved in muscle relaxation via calcium buffering ^5^. Unfortunately, the development of specific immunotherapeutics for preventing fish allergies is limited. As a result, prophylactic management of this allergy relies only on the elimination of fish products from the diet of sensitized patients ^4^. In salmonids, a single parvalbumin-beta1 protein was identified as an allergen in some patients ^4^. Conceptually, a simple way to suppress trout allergy is to inactivate the gene encoding parvb1. Nevertheless, one cannot rule out the possibility that the invalidation of parvb1 might prevent fish development and/or affect muscle physiology. Consistent with this remark, we observed that crossbreeding of two distinct trout (F1) lines carrying one null parvb1 allele did not lead to the generation of homozygous F2 mutants (Lebret *et al.*, unpublished data). Given that parvb1 exon 2 encodes an allergenic peptide ^4^, we aimed to generate Parvb1 alleles that specifically lack this exon. To this end, we attempted to perform exon 2 skipping and swapping by applying microhomology-mediated end-joining (MMEJ) knock-in, a process that facilitates the insertion of donor DNA flanked by a small region (approximately 25nt) homologous to the Crispr target site ^6, 7^.

## Methods

### Guide RNA (gRNA) design and synthesis

**Figure 1.**
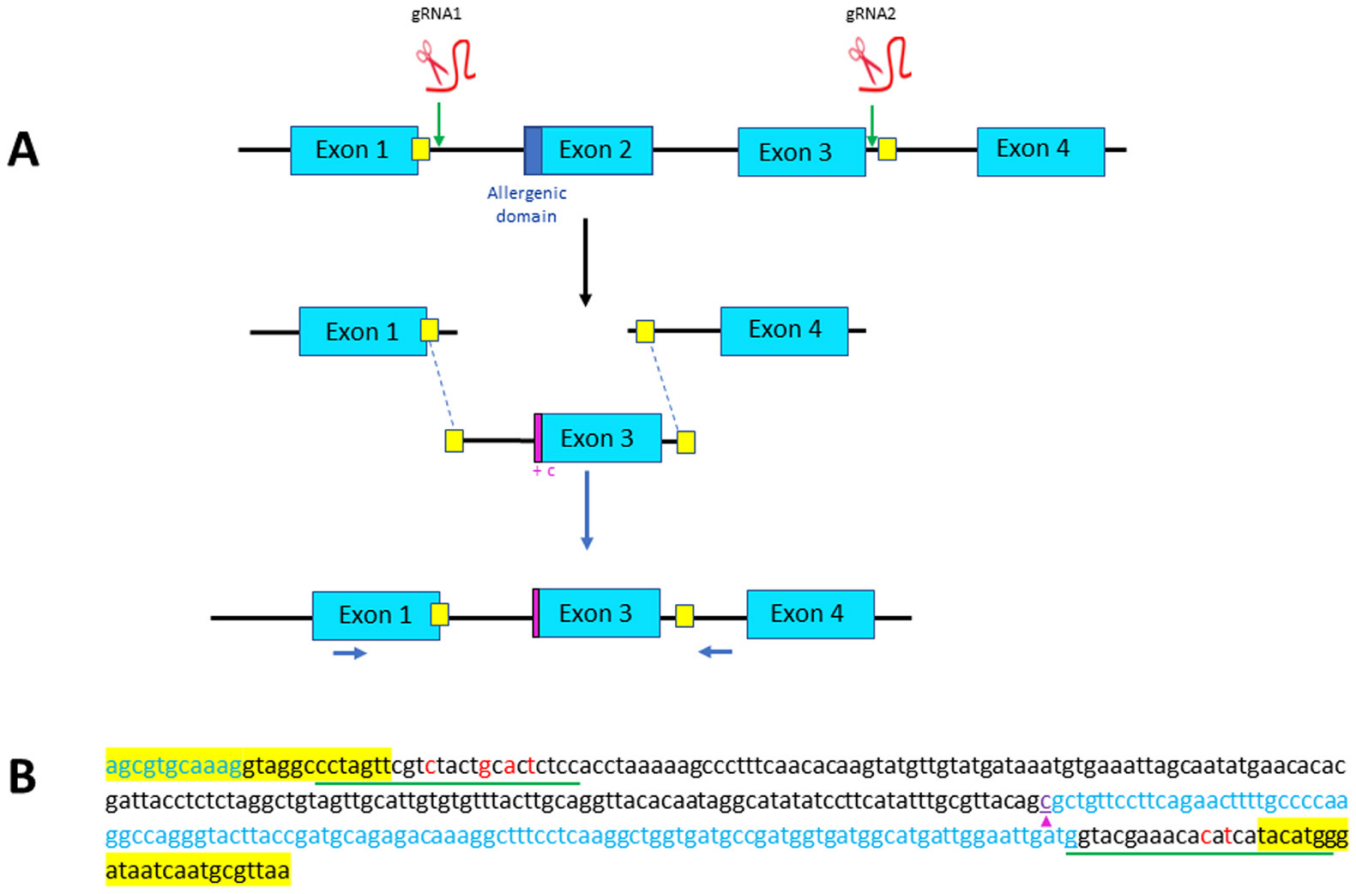
Strategy designed for Exon 2 skipping through microhomology-mediated end joining (MMEJ). ( **A**) Exons 2 and 3 of parvb1 locus are excised using two gRNAs, one (gRNA1) located in the intron between exons 1 and 2, and one (gRNA2) in the intron between exons 3 and 4. The two cutting-sites targeted by the gRNAs are flanked by short sequence (yellow squares) that are added as homology arm at the 5’ and 3’ ends of the donor DNA (containing only parvb1 exon 3) to be inserted. Position of primers used for PCR screening are indicated (blue arrows). ( **B**) Nucleotide sequence of the donor DNA used for exon skipping. The short homology arms are highlighted in yellow. The gRNA sites of the donor DNA (underlined in green) have been slightly modified (red letters) to prevent cas9-mediated re-cutting of the donor DNA after its integration into parvb1 locus. A cytosine (purple arrowhead) has been added at the 5’ extremity of exon 3 to keep the reading frame of exon 1.

The guide RNAs used were selected using the Crispor tool ( http://crispor.tefor.net/), which identifies gRNAs, evaluates potential off-targets, and predicts on-target activity. gRNAs and *cas9* mRNA were prepared as previously described ^8^. Briefly, the DR274 vector (Addgene #42250) containing the universal guide RNA sequence was linearized with Bsa1 and purified. PCR amplifications were performed using linearized DR274 as a template and specific primers for each sgRNA. Forward primers containing sgRNA target sequences (bold and underlined) between the T7 promoter sequence at the 5' end and the conserved tracrRNA domain sequence were as follows: forward primer (#site 1), 5' GAAATTAATACGACTCACTATA **GGAGGGGGAAGTAAACGAACT** GTTTTAGAGCTAGAAATAGCAAG-3'. Forward primer (#site 2): 5'-GAAATTAATACGACTCAC TATA **GGTACGAAACATAGCATACA**GTTTTAGAGCTAGAAATAGCAAG-3'; universal Reverse primer: 5'-AAAAGCACCGACTCGGTGCCACT-3'. Subsequently, the residual plasmid was digested using Dpn1. The final product was purified and used as the DNA template for transcription. sgRNAs were transcribed using the MegaShortScript T7 Transcription Kit (Ambion (cat. AM1354)) according to the manufacturer’s instructions. The double-stranded templates ( Figure 1B and Figure 4B) added to the injection mix for MMEJ Knock-In experiments were provided by GeneStrand Synthesis, Eurofin Genomics.

### Preparation of embryos and microinjection

Trout eggs were collected by gentle manual stripping under anesthesia (2-phenoxyethanol, 0.05%). The eggs were then fertilized by careful mixing with the sperm solution. For microinjection, a mixture containing gRNA (100ng/μl), cas9 mRNA (200ng/μl) and double-stranded donor DNA (10 ng/μl) was prepared and microinjected using a FemtoJet (Eppendorf) microinjector during a period of 2 to 6 h after egg fertilization (one cell stage prior to the first cleavage). Once injected, embryos were transferred to hatchery trays and incubated in running water (10°C) until first feeding (65 dpf). Subsequently, the fingerlings were transferred into indoor tanks.

### Screening for mutations

Genotyping was performed using genomic DNA extracted from the fin tissue of 70 dpf fingerlings. Specifically, fingerlings were anesthetized in 2-phenoxyethanol (0.5 ml/L) and a small part (2mm ^2^) of the caudal fin was clipped. Fin-clip samples were then incubated in 100 μl of 5% Chelex 100 Molecular Biology Grade resin (BioRad (cat.1421253)) and 50 μl of proteinase K solution (20mg/ml, Ambion (cat. AM2546)) for 2 h at 55°C and then for 10 min at 99°C with continuous stirring. After centrifugation at 5000 g for 10 min, the supernatant was collected and subsequently used for PCR. For the exon skipping experiment, PCR was performed using the forward primer 5’-CAATGCTCAATACCGAACGTTGA-3’ and the reverse primer 5’-AGAGGCAGTAGTGATGACCAG-3’ present in parvb1 exon1 and intron3 ( Figure 1A), respectively. For the exon swapping experiment, PCR was performed using the forward primer 5’-CAATGCTCAATACCGAACGTTGA-3’ present in parvb1 exon1, the reverse primer 5’-AGAGGCAGTAGTGATGACCAG-3’ present in parvb1 intron3 and the reverse primer 5’-TCCTCAATGAAGCCACTCTTG-3’ specifically present in parvb2 exon 2 ( Figure 4A). The PCR cycling conditions were as follows: 95°C for 5 min and 35 cycles of 95°C for 15 s, 60°C for 30s, and 72°C for 30 s. The reaction products were separated and visualized using agarose gel electrophoresis. PCR products of interest were sequenced using PCR primers provided by the Eurofins Genomic Sequencing Service ( https://www.eurofinsgenomics.eu).

## Results

### Exon 2 skipping

**Figure 2.**
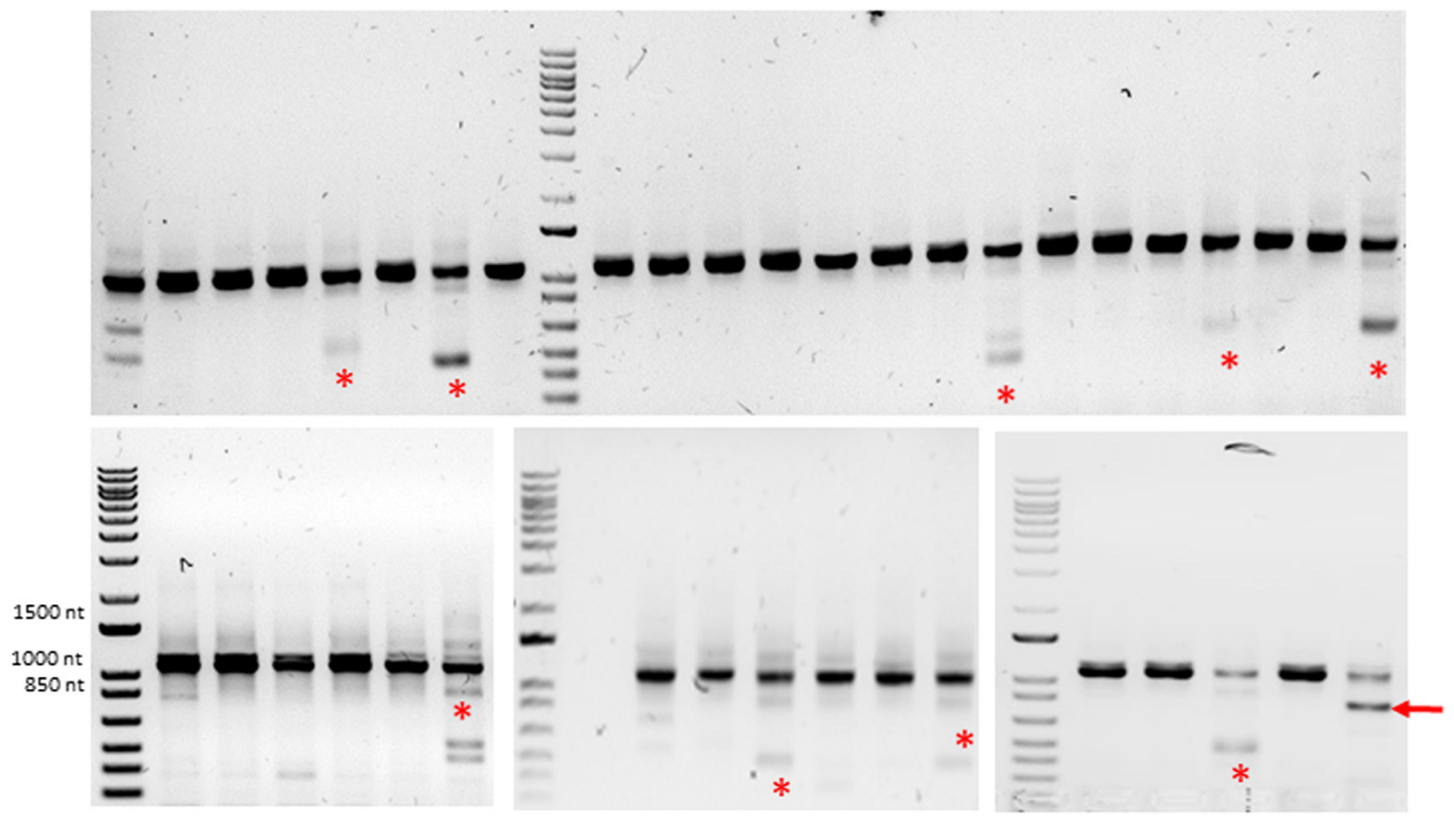
Exon skipping experiment: detection of Crispr/Cas9-introduced mutations by genotyping. Gel electrophoresis of several PCR products: truncated alleles of various sizes (red stars) were sometimes found in addition to the wild type allele; only one individual displayed an allele carrying a deletion of the expected size (red arrow).

**Figure 3.**
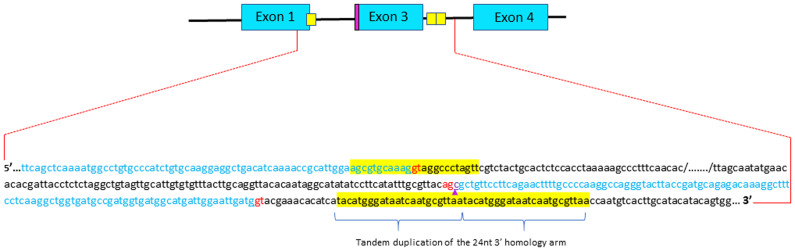
Sequencing analysis of integration junctions of the donor DNA into parvalbumin beta 1 locus in the animal displaying exon2 skipping. Short homology arms are highlighted in yellow. Although the donor DNA is correctly inserted, one can observed a tandem duplication of the 3’ short homology arm. The additional cytosine that kept the reading frame of exon 1 is outlined (purple arrow). Donor and acceptor splice sites are in red.

Parvb1 is composed of four exons ( Figure 1A). The 5’ subdomain of exon 2 encodes the allergenic peptide, whereas exons 3 and 4 encode the C-terminal EF-hand motif involved in calcium binding ^4^. Exons 1 and 3 of parvb1 are not in frame, so a single excision of exon 2 followed by NHEJ would not result in appropriate splicing of the remaining exons. Therefore, to perform correct exon 2 skipping, we sought to delete both exon 2 and exon 3 using appropriate guide RNAs ( Figure 1A), and to concomitantly elicit, at the cutting sites, the integration of an injected donor DNA containing parvb1 intron 1, exon 3 (with an additional nucleotide immediately following the splice acceptor site to maintain the reading frame of exon 1), and the 5’ domain of intron 3 ( Figure 1 A and B). The donor DNA was flanked by short homology arms for proper insertion through microhomology-mediated end joining (MMEJ). Out of approximately 600 fertilized eggs injected to achieve exon2 skipping, only 191 developed up to the fingerling stage (approximately 70 d post-fertilization). The fingerlings were genotyped using appropriate primers ( Figure 1A). In most cases, only one PCR band corresponding to the wild-type allele was found in fingerlings. Truncated alleles of various sizes were sometimes observed (10 fingerlings out of 191) in addition to the wild-type allele ( Figure 2). Only one individual exhibited a truncated allele corresponding to the correct insertion of donor DNA ( Figure 2). Sequencing of this allele confirmed the correct insertion of donor DNA but also revealed a tandem duplication of the 3’ microhomology arm in intron 3 ( Figure 3). Given its position within the gene, it is unlikely that this tandem duplication would affect the processing of the parvb1 primary transcript. Unfortunately, the premature death of fingerlings carrying this mutation divested the F1 generation for further phenotyping. Overall, this study shows that microhomology-mediated end joining can be used to delete exon 2 of parvb1, although the success rate was very low.

### Exon 2 swapping

**Figure 4.**
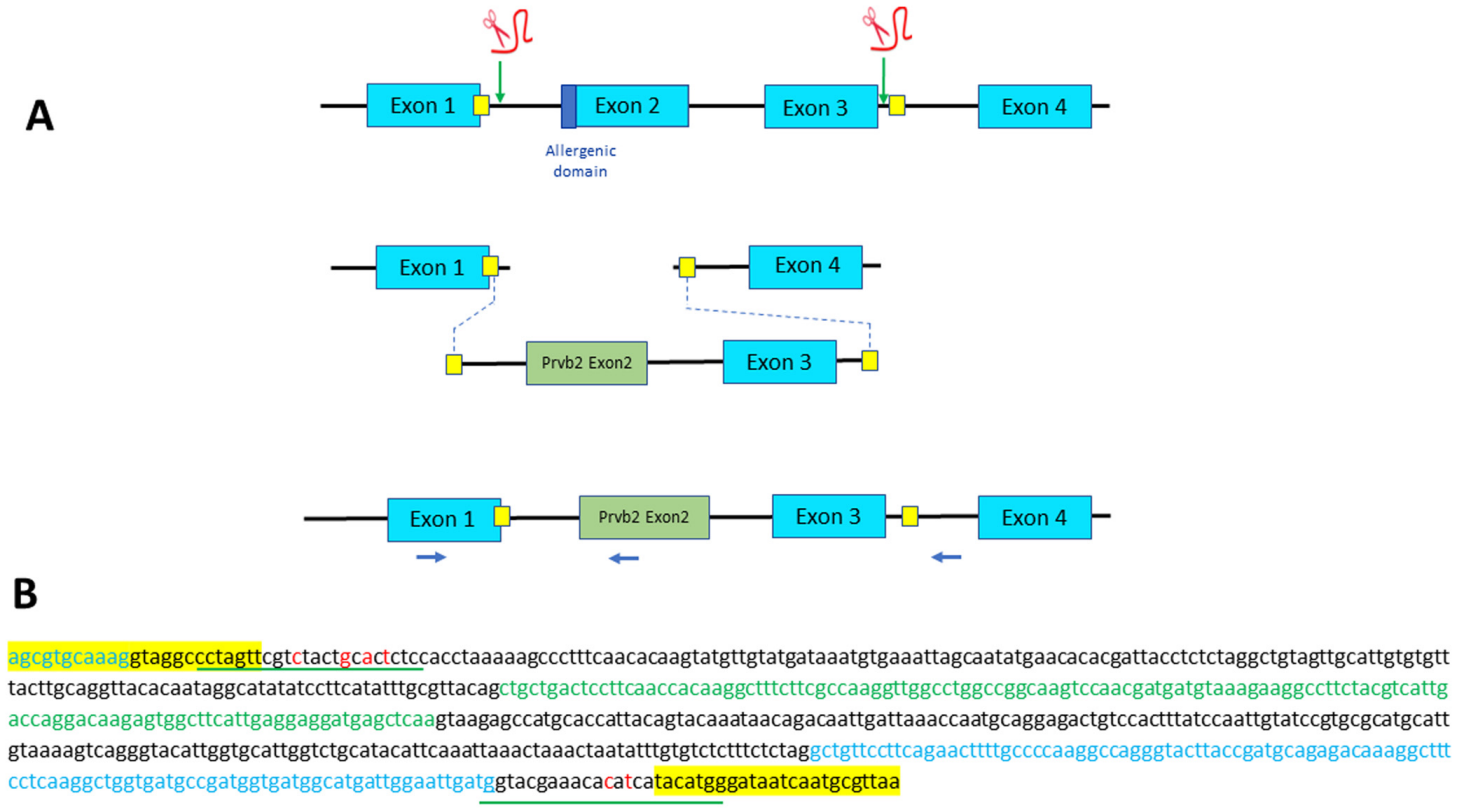
Strategy designed for Exon 2 swapping through microhomology-mediated end joining (MMEJ). ( **A**) Exons 2 and 3 of parvb1 locus are excised using two gRNAs, one located in the intron 5’ to exon 2 and one in the intron between exons 3 and 4. The two cutting-sites targeted by the gRNAs are flanked by a short sequence (yellow squares) that are added as homology arm at the 5’ and 3’ ends of the donor DNA (containing parvb2 exon 2 and parvb1 exon 3) to be inserted. Position of primers used for PCR screening are indicated (blue arrows). ( **B**) Nucleotide sequence of the donor DNA used for exon 2 swapping. Parvb2 Exon 2 and parvb1 exon 3 are in green and blue letters, respectively. Short homology arms are highlighted in yellow. The gRNA sites (underlined in red) have been slightly modified (red letters) to prevent cas9-mediated re-cutting of the donor DNA after its integration into parvalbumin beta 1 locus.

To replace parvb1 exon2 with parvb2 exon2, which does not encode the allergenic peptide, we excised exons 2 and 3 using the same gRNAs employed for parvb1 Exon2 skipping ( Figure 1A). Knock- In was carried out using donor DNA containing parvb1 intron 1, parvb2 exon 2, parvb1 intron 2, parvb1 exon 3, and the 5’ domain of parvb1 intron 3 ( Figure 4 A and B). Short homology arms were added at the extremities of the donor DNA to achieve proper insertion through microhomology-mediated end joining (MMEJ) ( Figure 4B). Approximately 500 fertilized eggs were injected, 157 of which developed up to the fingerling stage. These fingerlings were genotyped, but none of them were found to display the correct donor DNA insertion into the parvb1 locus, as shown after genotyping using an internal parvb2 specific primer ( Figure 4A). Interestingly, in contrast to our initial exon-skipping experiment, no truncated junk alleles were observed. This second part of our study confirms an apparently intrinsically low incidence for the targeted integration of DNA templates through microhomology-mediated end joining in trout.

## Discussion

Parvalbumin beta-1 is a major allergen in the trout. Therefore, the invalidation of parvb1 encoding gene could prevent allergic reactions in sensitive persons. However, one can rule out the possibility that knockout of the gene encoding parvb1 could have deleterious developmental and/or physiological effects on the whole animal. In keeping with this point, we were unable to generate homozygous mutants after crossing two distinct F1 lines carrying one null allele (Lebret, unpublished work). Hence, precise engineering that allows the production of active parvb1 specifically deprived of the allergenic domain remains the only possible alternative strategy to suppress fish allergenicity. Given that the allergenic domain of parvb1 is encoded by exon2, our editing strategy was to use the Crispr/cas9 system to generate F0 trout carrying either an allele lacking exon 2 (exon skipping) or displaying the replacement of parvb1 exon 2 with that of parvalbumin beta-2 (parvb2), which does not encode the allergenic peptide (exon swapping). For this purpose, we tested the possibility of inserting specific donor DNA through the microhomology-mediated end-joining pathway (MMEJ). (MMEJ)-based methods are designed to integrate donor DNA into a targeted cutting site of the genome, provided that the donor DNA contains, at its 5’ and 3’ ends, short arms that are homologous to the sequences flanking the targeted cutting site ^6, 7^. Building on the work by Paquet *et al.* ^9^ and feedback from preliminary experiments on medaka (Herpin, unpublished results), we introduced silent CRISPR/cas9 blocking mutations in the guide RNA sequence adjacent to the microhomology arms to prevent re-cutting of the target sequence once the donor DNA has been integrated into the genome. In our study, only one individual (out of 191) displayed exon 2 exon skipping through microhomology-mediated end-joining Knock-In, and not a single animal (out of 157) exhibited exon swapping. The efficiency of the procedure was disappointingly low and clearly below our expectations when compared with the success rate obtained in medaka. Some success has been reported regarding Crispr/cas9-mediated knock-in in farmed fish, such as carp ( *Laboeo rohita*) ^10^ and salmon ( *Salmo salar L*.) ^11^. Nevertheless, in the first report ^10^, the two homology arms of the donor DNA to be inserted were considerably longer (800 nt) than those used for MMEJ (20-30nt) and in the second report ^11^, the sequence to be inserted through microhomology-mediated end-joining Knock-In was very short (<100 nucleotides). Therefore, gene editing to insert any desired sequence in farmed fish genomes needs to be carefully considered. Not only is knock-in efficiency obviously uncertain, but germinal mosaicism in the F0 generation is also important. Being often long, the generation time of farmed fish species (two years for trout) might be an obstacle for producing heterozygous F1 and homozygous F2 lines. Regarding knock-in efficiency, improvements can be made by testing templates of different lengths. The length of the homologous arms, as well as the use of asymmetric donor DNA containing homology arms of different sizes at the 5’ and 3’ ends, are also parameters that might impact KI efficiency ^12^. Chemical intervention, blocking the NHEJ pathway, and promoting the MMEJ pathway, such as NU7441 ^13^, might also be a suitable approach. Beyond the fact that effective harnessing of MMEJ remains challenging ^14^, it is important to point out that genome editing using donor DNA is likely to lead, at some point, to uncontrolled and random insertion of the donor DNA into the host genome. To illustrate this aspect, it is worth noting that 60% of trout embryos injected with a donor DNA containing a myosin promoter coupled with GFP cDNA gave rise to F0 individuals displaying mosaic GFP fluorescence in skeletal muscle ^15^. Therefore, in addition to potential off-target mutations, animals edited by knock-in are likely to display high rates of unintentional transgene insertions. This highlights the necessity of conducting careful whole-genome sequencing of knock-In farmed animals to limit introgression of the template inserted in non-targeted loci during successive crossings. This implies that gene editing must be performed solely in animals in which the genome has been fully sequenced and annotated. In July 2018, the Court of Justice of the European Union delivered a judgement in which it held that organisms modified by genome-editing techniques are genetically modified organisms (GMOs) and fall within the scope of EU legislation on GMOs (see footnote: infocuria Jurisprudence). This judgement, which makes no difference between a gene-edited animal displaying a single base pair substitution and a transgenic animal carrying exotic DNA, is highly disputed, as recently reported by a European commission study concluding that the present legislation is not suitable for new genomic techniques (NGT) and needs some adaptations to follow rapid scientific and technological progress (see footnote: New genomic techniques, European commission study and first reactions). In any event, we believe that revision of the law determining the legal status of gene-edited animals would gain much from a basic distinction between those edited using donor DNA (HDR, MMEJ, etc.) and those edited without donor DNA (NHEJ). The former is potentially transgenic if one posits that the insertion(s) of any donor DNA outside of its natural locus falls under transgenesis. Whether the Crispr/cas9-mediated KI gene should be developed in aquaculture breeding programs depends on consumer acceptance as well as government regulation. There have been lively debates on this point, notably in the European parliament. We believe that our data can contribute to feed debates regarding the feasibility and limitations of CRISPR-mediated knock-Ins in farmed fish, as well as future regulatory actions for their generation and use.

## Ethical statement

Fish care and sampling were conducted at the INRAE UR1037 fish facilities (DDSP approval D35-238-6) in strict accordance with the European policies and the guidelines of the National Legislation on Animal Care and Use Ethical Committee (Decree No.2013–118, February 1st, 2013; European Directive 2010–63, September 22, 2010). Experimental protocols involving animals were approved by the “Comité Rennais d’éthique pour l’expérimentation animale’ (CREEA, ref. APAFIS #31048-2021041509574912V3).

## Animal ethics

All efforts were made to prevent fish suffering. In particular, trout eggs used for microinjection were collected by gentle manual stripping of mature anesthetised females. Also, regarding fingerling genotyping, fin tissue sampling was carried out on anesthetised animals according to current standards.

## Data Availability

NCBI GenBank: Oncorhynchus mykiss parvb1 artificial allele lacking exon 2, partial sequence. Accession number PQ067334, https://www.ncbi.nlm.nih.gov/search/all/?term=PQ067334 Zenodo: - Microhomology-mediated end-joining Knock-In approaches to delete the allergenic domain of trout parvalbumin beta-1. Preliminary results in F0 animals and feedback, https://zenodo.org/records/13804556 ^16^. DOI: http://dx.doi.org/10.5281/zenodo.13804556 This project contains the following data: Pages of the laboratory notebook containing PCR gel photographs. Data are available under the terms of the Creative Commons Attribution 4.0 International license (CC-BY 4.0) https://creativecommons.org/licenses/by/4.0/ This study is reported in accordance with ARRIVE guidelines: DOI: http://dx.doi.org/10.5281/zenodo.13803707 ^17^ Data are available under the terms of the Creative Commons Attribution 4.0 International license (CC-BY 4.0)

## Footnotes

Infocuria jurisprudence :
https://curia.europa.eu/juris/document/document.jsf;jsessionid=C53743C718B8F4A96ADB9AE3840F765A?text=&docid=204387&pageIndex=0&doclang=EN&mode=req&dir=&occ=first&part=1&cid=6190527

New genomic techniques, European Commission study and first reactions:
https://www.europarl.europa.eu/RegData/etudes/BRIE/2021/698760/EPRS_BRI(2021)698760_EN.pdf
