# Supplementary material for: Microhomology-mediated end-joining Knock-In approaches to delete the allergenic domain of trout parvalbumin beta-1. Preliminary results in F0 animals and feedback
Source: Open Res Eur. 2025 Feb 25;4:235. Originally published 2024 Oct 25. [Version 2] doi: 10.12688/openreseurope.18223.2 (PMC11876799; doi:10.12688/openreseurope.18223.2)
Supplement: Peer review history

# Reviewer report: Reviewer response for version 2

**DOI:** 10.21956/openreseurope.21305.r51571

**Published:** 2025-03-18

**Authors:** Joana Costa

**Affiliations:** 1 Faculty of Pharmacy, University of Porto, Porto, Porto District, Portugal; 2 REQUIMTE LAQV Porto, Porto, Portugal

The authors have addressed my concerns in this revision. I have no more comments

Is the study design appropriate and does the work have academic merit?

Partly

Is the work clearly and accurately presented and does it cite the current literature?

Yes

If applicable, is the statistical analysis and its interpretation appropriate?

Not applicable

Are all the source data underlying the results available to ensure full reproducibility?

Partly

Are the conclusions drawn adequately supported by the results?

Yes

Are sufficient details of methods and analysis provided to allow replication by others?

Partly

Reviewer Expertise:

Food allergy and immunology, Food allergen characterization, food allergen detection (DNA-and protein-based methods), GMO detection.

I confirm that I have read this submission and believe that I have an appropriate level of expertise to confirm that it is of an acceptable scientific standard.

# Reviewer report: Reviewer response for version 2

**DOI:** 10.21956/openreseurope.21305.r51569

**Published:** 2025-03-03

**Authors:** Qing Xiong

**Affiliations:** 1 The Hong Kong Polytechnic University, Hong Kong, Hong Kong

The authors have addressed my concerns in this revision. I have no more comments.

Is the study design appropriate and does the work have academic merit?

Partly

Is the work clearly and accurately presented and does it cite the current literature?

Yes

If applicable, is the statistical analysis and its interpretation appropriate?

I cannot comment. A qualified statistician is required.

Are all the source data underlying the results available to ensure full reproducibility?

Partly

Are the conclusions drawn adequately supported by the results?

Yes

Are sufficient details of methods and analysis provided to allow replication by others?

Partly

Reviewer Expertise:

Allergy and infectious diseases, Genomics and bioinformatics

I confirm that I have read this submission and believe that I have an appropriate level of expertise to confirm that it is of an acceptable scientific standard.

# Reviewer report: Reviewer response for version 1

**DOI:** 10.21956/openreseurope.19693.r47220

**Published:** 2025-01-09

**Authors:** Joana Costa

**Affiliations:** 1 Faculty of Pharmacy, University of Porto, Porto, Porto District, Portugal; 2 REQUIMTE LAQV Porto, Porto, Portugal

The manuscript describes the application of gene editing techniques to decrease the allergenicity of trout by deleting the exon that codes for the allergenic peptide of parvb1 and substitute it by another from parvb2 encoding a non-allergenic peptide. The idea is well presented, and the experiments seem well designed. Additionally, the manuscript details the main constraints encountered in this study, such as the size of the sequence to be inserted. Another drawback regards the generation time of farmed fishes (>2 years for the trout). Still, some concerns may be raised based on some missing gaps, which could be addressed.

 Comments:

 Materials and Methods – this section would benefit from more information, namely regarding PCR performance, such as mix conditions and equipment. Scientific name of the trout should also be presented.

 Results – It would be important to conduct an allergenicity/IgE-binding assessment, in order to prove that the genetic modification was efficient in decreasing the expression of allergenic parvalbumin, which could be done by extracting parvalbumin from F0 and wild type trout and test them against sera of fish allergic patients. This would allow to better conclude if the main objective of this manuscript would have been accomplished or not. If not performed, this aspect should be at least discussed as a fundamental step (proof of concept) for attaining a fish with potentially reduced allergenicity.

Is the study design appropriate and does the work have academic merit?

Partly

Is the work clearly and accurately presented and does it cite the current literature?

Yes

If applicable, is the statistical analysis and its interpretation appropriate?

Not applicable

Are all the source data underlying the results available to ensure full reproducibility?

Partly

Are the conclusions drawn adequately supported by the results?

Yes

Are sufficient details of methods and analysis provided to allow replication by others?

Partly

Reviewer Expertise:

Food allergy and immunology, Food allergen characterization, food allergen detection (DNA-and protein-based methods), GMO detection.

I confirm that I have read this submission and believe that I have an appropriate level of expertise to confirm that it is of an acceptable scientific standard, however I have significant reservations, as outlined above.

# Response

**Published:** 2025-02-13

**Authors:** Pierre-yves Rescan

**Affiliations:** physiology, Institut National de Recherche pour l'Agriculture l'Alimentation et l'Environnement Centre Bretagne-Normandie, Rennes, Brittany, France

Dear Dr Joana Costa

 Thank you for your support in reviewing our manuscript. Here, we present our responses to your valuable comments.  

**The manuscript describes the application of gene editing techniques to decrease the allergenicity of trout by deleting the exon that codes for the allergenic peptide of parvb1 and substitute it by another from parvb2 encoding a non-allergenic peptide. The idea is well presented, and the experiments seem well designed. Additionally, the manuscript details the main constraints encountered in this study, such as the size of the sequence to be inserted. Another drawback regards the generation time of farmed fishes (>2 years for the trout). Still, some concerns may be raised based on some missing gaps, which could be addressed.**

 Comments:

**Materials and Methods – this section would benefit from more information, namely regarding PCR performance, such as mix conditions and equipment. Scientific name of the trout should also be presented.**

 Answer: Further details regarding PCR conditions and equipment are provided in the revised version. Also was specified the scientific name of the trout : *Oncorhynchus mykiss* (Walbaum, 1792)

**Results – It would be important to conduct an allergenicity/IgE-binding assessment, in order to prove that the genetic modification was efficient in decreasing the expression of allergenic parvalbumin, which could be done by extracting parvalbumin from F0 and wild type trout and test them against sera of fish allergic patients. This would allow to better conclude if the main objective of this manuscript would have been accomplished or not. If not performed, this aspect should be at least discussed as a fundamental step (proof of concept) for attaining a fish with potentially reduced allergenicity.**

 Answer. We agree that our manuscript deserves further explanations. Of course, our ultimate goal is to assess allergenicity of trout carrying null or modified parvb1 alleles testing sera from allergic patients. Towards this aim a collaboration was planned with Anette Kuehn (laboratory of immunogenetics and allergology, Luxembourg). Given that allergenicity is triggered by traces of allergens we believe that it is preferable to carrying out allergenicity tests on F2 (parvb1-/-) trout completely depleted of parvb1 and not in F0 trout that are likely to produce significant amount of parvb1. Unfortunately, as indicated in our manuscript, we were unable to generate homozygous null mutant after crossing two distinct F1 lines carrying one null allele.  Therefore, the only way to continue our project was to specifically delete the allergenic domain of parvb1 gene using Knock-in. Unexpectedly, the MMEJ KI success rate was at the end found to be very low. As such, the only (and deliberately limited) aim the of the present manuscript is to point out the potential difficulties we encountered with MMEJ Knock In and to discuss new genomic techniques in aquaculture in the context of the lively debates, notably in the European parliament, regarding the feasibility and limitations of CRISPR-mediated Knock-Ins in farmed fish. In keeping with your comment, we have added, in the discussion section, a paragraph presenting future research axis in relation with possible evolutions of our research .

# Reviewer report: Reviewer response for version 1

**DOI:** 10.21956/openreseurope.19693.r47708

**Published:** 2024-12-04

**Authors:** Qing Xiong

**Affiliations:** 1 The Hong Kong Polytechnic University, Hong Kong, Hong Kong

As preliminary results, this manuscript is promising but could be improved in several aspects:

1. The use of the common name "trout" is not sufficiently precise. The authors should clearly define the specific species used in this study, as there are many species referred to as trout.
2. Building on the previous point, it would be advantageous to conduct whole genome and transcriptome sequencing for the model organism used in this study. This is important because fish species often have multiple parvalbumin paralogs (refer 1). Whole genome sequencing is also powerful to detect the possible off-target effects after the knock-in experiment.
3. Additionally, given the numerous parvalbumin paralogs in trout, it would be beneficial to compare transcriptome changes among these paralogs before and after the knock-in experiment.

Lastly, while I believe that the authors will assess changes in allergenicity, I strongly recommend that they consider all parvalbumin paralogs. This is directly related to allergenic potential and where off-target effects may occur.

Is the study design appropriate and does the work have academic merit?

Partly

Is the work clearly and accurately presented and does it cite the current literature?

Yes

If applicable, is the statistical analysis and its interpretation appropriate?

I cannot comment. A qualified statistician is required.

Are all the source data underlying the results available to ensure full reproducibility?

Partly

Are the conclusions drawn adequately supported by the results?

Yes

Are sufficient details of methods and analysis provided to allow replication by others?

Partly

Reviewer Expertise:

Allergy and infectious diseases, Genomics and bioinformatics

I confirm that I have read this submission and believe that I have an appropriate level of expertise to confirm that it is of an acceptable scientific standard, however I have significant reservations, as outlined above.

## References

[1] : Comparative analysis of asian carps parvalbumin reveals the divergence pattern of major fish allergen. *Asian Pac J Allergy Immunol* .2024; 10.12932/AP-200823-1673 10.12932/AP-200823-1673 38710642

# Response

**Published:** 2025-02-13

**Authors:** Pierre-yves Rescan

**Affiliations:** physiology, Institut National de Recherche pour l'Agriculture l'Alimentation et l'Environnement Centre Bretagne-Normandie, Rennes, Brittany, France

Dear Dr Qing Xiong,

 Thank you for your effort in handling our manuscript. The manuscript was revised with attention to your recommendations. Please find below our response to the comments.    

**Reviewer Comments:**

 As preliminary results, this manuscript is promising but could be improved in several aspects:

 1. The use of the common name "trout" is not sufficiently precise. The authors should clearly define the specific species used in this study, as there are many species referred to as trout.

**Answer**. We fully agree. The trout species we used for this study was *Oncorhynchus mykiss *(Walbaum, 1792 *)*. This information is now provided in the “Methods” section.

**Reviewer Comments:**

 2. Building on the previous point, it would be advantageous to conduct whole genome and transcriptome sequencing for the model organism used in this study. This is important because fish species often have multiple parvalbumin paralogs (refer 1). Whole genome sequencing is also powerful to detect the possible off-target effects after the knock-in experiment.

**Answer. **Oncorhynchus mykiss genome has been sequenced, assembled and annotated (Berthelot et al., 2014). As such, we were able to identify gRNAs specifically targeting Parvb1, evaluate potential off-target and predicts on-target activity. This information about Oncorhynchus mykiss genome knowledge has been added to the “methods” section.

**Reviewer Comments:**

 3. Additionally, given the numerous parvalbumin paralogs in trout, it would be beneficial to compare transcriptome changes among these paralogs before and after the knock-in experiment.

**Answer.** We agree that it would be of interest to compare transcriptome changes before and after the knock-in experiment. But, for this purpose, it is necessary to produce homozygous F2 mutants. Unfortunately, although KI procedure could be considered as technically trivial, the success rate using MMEJ KI experiments was found to be very low. Therefore, the ambition of our manuscript was only to indicate that complex gene editing strategies, at least in farmed fish, definitely need further improvements before being routinely employed as popular procedure for engineering desirable phenotypic traits.  

**Reviewer Comments:**

 4.  Lastly, while I believe that the authors will assess changes in allergenicity, I strongly recommend that they consider all parvalbumin paralogs. This is directly related to allergenic potential and where off-target effects may occur.

**Answer.** Indeed, different parvalbumin isoforms and isoallergens may be present in the same fish. Nevertheless, only a single trout parvb1 isoform was identified as allergen in specific patients (Kuehn et al., 2014). Therefore, invalidating or modifying this isoform could prevent allergy in these specific patients.

 Nevertheless, we agree that if changes in allergenicity are evidenced after parvb1 gene editing (coll. A. Kuehn), then it will be important to check that they are not related to undesirable off-targets affecting other parvalbumin paralogs.

# Reviewer report: Reviewer response for version 1

**DOI:** 10.21956/openreseurope.19693.r45526

**Published:** 2024-11-11

**Authors:** Jitendra Kumar Sundaray

**Affiliations:** 1 ICAR-Central Institute of Freshwater Aquaculture, Bhubaneswar-751002, Odisha,, India

- The MS does not provide adequate information on Microhomology-Mediated End Joining (MMEJ).  
- In the exon skipping experiment, it was noted that only 10 indels were detected out of 191 samples. , This is a low efficiency, and the paper could have added an explanation about the same.( such as..gRNA efficiency)
- In the exon swapping experiment, exon 2 of parvb 2 have been inserted- It would be useful to clarify whether Exon 2 encodes any critical functional domains  -Could have provided more insights.
- The same set of gRNAs is used in both the exon skipping and exon swapping experiments, yet there were no observed indels in the exon swapping experiment.What could be the possible reasons?   
- In Figure 2 (Genotyping image), it would be helpful to include wild-type trout DNA samples as a baseline for comparison.  

Is the study design appropriate and does the work have academic merit?

Yes

Is the work clearly and accurately presented and does it cite the current literature?

Yes

If applicable, is the statistical analysis and its interpretation appropriate?

Not applicable

Are all the source data underlying the results available to ensure full reproducibility?

Yes

Are the conclusions drawn adequately supported by the results?

Partly

Are sufficient details of methods and analysis provided to allow replication by others?

Partly

Reviewer Expertise:

Fish Genetics,  Microbiome and Genome editing

I confirm that I have read this submission and believe that I have an appropriate level of expertise to confirm that it is of an acceptable scientific standard, however I have significant reservations, as outlined above.

# Response

**Published:** 2025-02-13

**Authors:** Pierre-yves Rescan

**Affiliations:** physiology, Institut National de Recherche pour l'Agriculture l'Alimentation et l'Environnement Centre Bretagne-Normandie, Rennes, Brittany, France

11 Nov 2024 Version 1

 Jitendra Kumar Sundaray , ICAR-Central Institute of Freshwater Aquaculture, Bhubaneswar-751002, Odisha,, India

 Dear Dr Jitendra Kumar Sundaray Thank you for your valuable comments on our manuscript. Here, we present our answers to your comments.  

- The MS does not provide adequate information on Microhomology-Mediated End Joining (MMEJ).  
- In the exon skipping experiment, it was noted that only 10 indels were detected out of 191 samples. , This is a low efficiency, and the paper could have added an explanation about the same.( such as..gRNA efficiency)

Answer. This point is important and, indeed, deserves further clarifications. The efficiency of gRNA1 and gRNA2 has been evidenced during preliminary experiments aiming to select among several gRNAs (12 in all…) those which displayed activity as shown by sequencing of PCR products. So, both RNAg1 and RNAg2 recurrently elicit indels when injected individually in zygote. Our genotyping based on gel electrophoresis of PCR products was appropriate to uncover alleles resulting from Parvb1 exon2 skipping or swapping but not alleles with small (<10 nucleotides) indels. So, it is clear that the use of gel electrophoresis alone underestimated efficiency of RNAg1 and RNAg2 after Exon skipping and swapping experiment. A sequencing of all PCR products would have clarified this point, but we did not systematically perform PCR products sequencing. In the revised version we added these essential considerations to the “Method” section.  

- In the exon swapping experiment, exon 2 of parvb 2 have been inserted- It would be useful to clarify whether Exon 2 encodes any critical functional domains -Could have provided more insights.

Answer. Indeed, we should have developed this point. Parvalbumins contain specific EF-hands motifs which are involved in the binding of calcium. Exon2 partly encodes one of the two EF-hands motifs present in parvb1 and parvb2 proteins. Replacing parvb1 exon2 with parvb2 exon2 that does not encode the allergenic peptide opened the interesting possibility of producing a modified parvb1 protein possessing the two EF-hand motifs but lacking the allergenic peptide. These information have been added at the beginning of the ‘Results” section (paragraph “Exon2 skipping”).

- The same set of gRNAs is used in both the exon skipping and exon swapping experiments, yet there were no observed indels in the exon swapping experiment. What could be the possible reasons?   

Answer: Again, alleles with small indels were likely to be produced in the exon swapping experiment as for exon skipping experiment using RNAg1 and RNAg2, but these indels are not detected using gel electrophoresis of PCR products (see above). Regarding the complete absence of proper insertion of the donor DNA used for exon swapping we can hypothesize that it relates to the greater length of the donor DNA used for exon swapping compared to that used for exon skipping.  

- In Figure 2 (Genotyping image), it would be helpful to include wild-type trout DNA samples as a baseline for comparison. 

Answer: Figure 2 was modified to include wild type DNA samples as requested.    

 Is the work clearly and accurately presented and does it cite the current literature?

 Yes

 Is the study design appropriate and does the work have academic merit?

 Yes

 Are sufficient details of methods and analysis provided to allow replication by others?

 Partly

 If applicable, is the statistical analysis and its interpretation appropriate?

 Not applicable

 Are all the source data underlying the results available to ensure full reproducibility?

 Yes

 Are the conclusions drawn adequately supported by the results?

 Partly

**Competing Interests** No competing interests were disclosed.

**Reviewer Expertise** Fish Genetics,  Microbiome and Genome editing

*I confirm that I have read this submission and believe that I have an appropriate level of expertise to confirm that it is of an acceptable scientific standard, however I have significant reservations, as outlined above.*
